# Mitigating challenges and expanding the future of vascular tissue engineering—are we there yet?

**DOI:** 10.3389/fphys.2022.1079421

**Published:** 2023-01-04

**Authors:** Adeeba Shakeel, Peter R. Corridon

**Affiliations:** ^1^ Department of Immunology and Physiology, College of Medicine and Health Sciences, Khalifa University, Abu Dhabi, United Arab Emirates; ^2^ Biomedical Engineering, Healthcare Engineering Innovation Center, Khalifa University, Abu Dhabi, United Arab Emirates; ^3^ Center for Biotechnology, Khalifa University, Abu Dhabi, United Arab Emirates

**Keywords:** transplantation, vascular tissue engineering, tissue engineered vascular grafts (TEVG), scaffold, vascular graft

## 1 Introduction

Atherosclerosis is still a significant cause of death in western societies. The leading cause of this cardiovascular disease is lipid accumulation and inflammation of the large arteries, which may lead to clinical complications such as arterial thrombosis, myocardial infarction, and ischemic stroke (Xenotransplantation, 1997). Drugs are usually the first treatment choice, even in the late stages of atherosclerosis. Sometimes, more aggressive treatment like Coronary artery bypass surgery (CABG) is needed. CABGs are performed by harvesting a vessel from the patient, but the patient undergoing two surgeries only added to their comorbidities. However, long-term results after CABG depend not only on the completeness of revascularization and the initial severity of coronary and myocardial lesions but also on comorbidities like diabetes mellitus, arterial hypertension, and pulmonary and renal disorders. Moreover, the limited availability of autografts was soon realized, and new options proposed by tissue engineering were started exploring to design synthetic grafts (Corridon et al., 2013).

**FIGURE 1:**
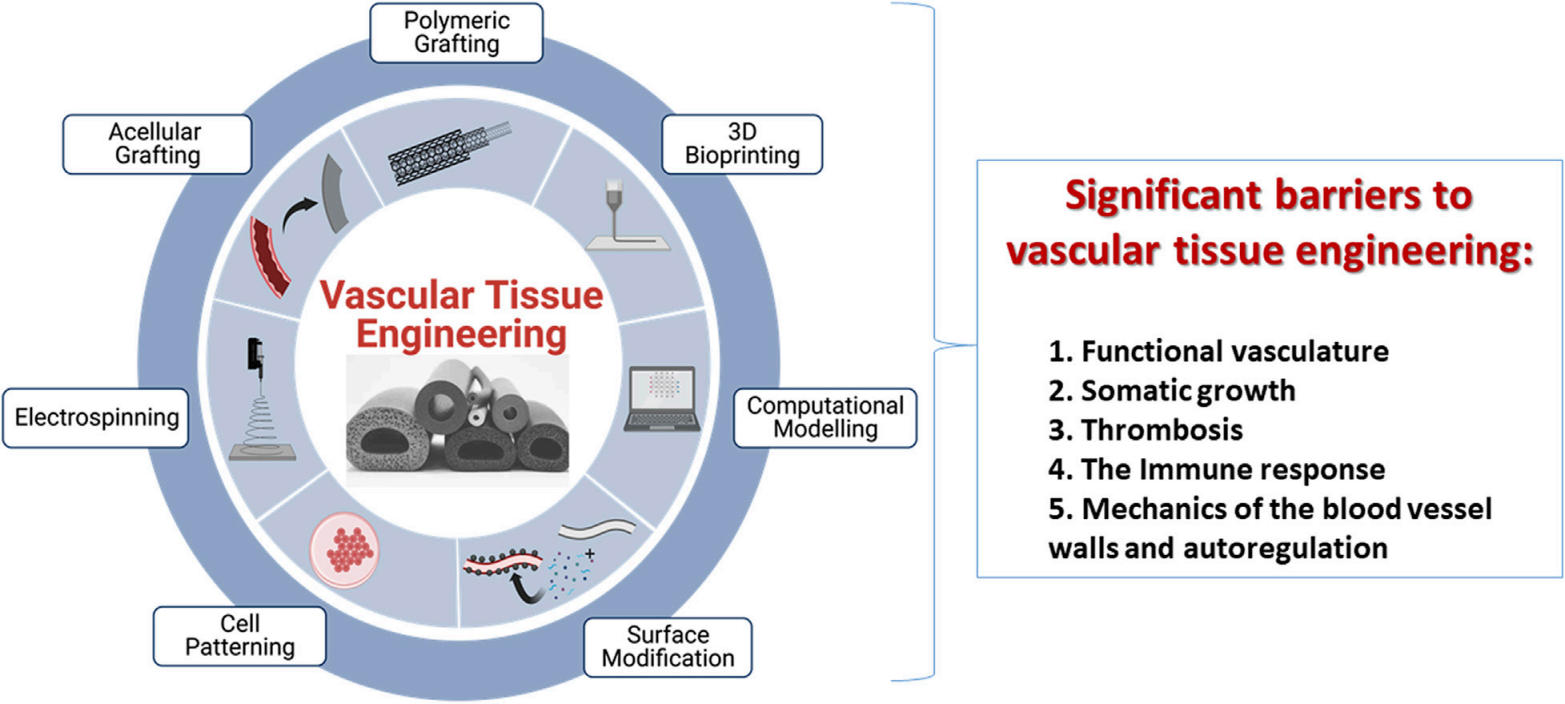
An illustration of current techniques, including polymeric grafting, 3D bioprinting, computational modeling, surface modification, cell patterning, electrospinning, and acellular grafting, used to create tissue-engineered vascular grafts with promising remodeling capabilities and limitations that restrict their viability post-transplantation. These limitations are a lack of functional vasculature, an inability to support somatic growth, detrimental induction of thrombosis post-transplantation, immune responses and biomechanics of the vessel wall.

Vascular tissue engineering (VTE) is focused on constructing vessels using different biomaterials, cell sources, biomolecules, and mechanical stimuli that can function in physiological environments (Lovett et al., 2009; Pradeep et al., 2019; Wang et al., 2022a). Such vessels replace non-functional vascular compartments and generate networks within bio (artificial) scaffolds. Pioneering efforts in this field date back to 1950, when artificial vascular grafts made from synthetic polymer materials were used to replace occluded arterial vessels (Song et al., 2018). It was perceived that biomaterials could support microvascular function showing great potential in accelerating the transition away from xenogenic materials for clinical application. For example, Li et al. (2017) developed a hyaluronic acid-based hydrogel chemically modified with fibronectin motifs that promote EC binding of α3/α5 b1 integrins, resulting in better vascularization to a non-modified hydrogel in a mouse stroke model. Similarly, advances in nanotechnology can bring additional functionality to vascular scaffolds, optimize internal vascular graft surface, reduce early thrombosis and inflammatory responses, and even direct the differentiation of stem cells into the vascular cell phenotype (Mironov et al., 2008; Wang et al., 2022a). However, two hurdles have persisted: achieving optimal vascularization in tissue-engineered scaffolds (Masson-Meyers and Tayebi, 2021; Corridon, 2022) and creating viable bioartificial vascular grafts (Corridon et al., 2017; Corridon, 2021; Wang et al., 2022b). Several techniques have been presented to address these challenges, Figure 1, which include decellularization, 3D bioprinting, and electrospinning (Neishabouri et al., 2022). These solutions have focused on ensuring that implantable biomimetic conduits, which exist as standalone grafts or those residing within more complex structures, have a healthy and adequate blood supply (Sarker et al., 2018; Devillard and Marquette, 2021). This essential characteristic is required to support gas diffusion, nutrient supply, and removal of waste that can sustain tissues and organs. However, integrating individual grafts into existing vascular networks represents a significant challenge (Traore and George, 2017; Meng et al., 2021; Blume et al., 2022). Specifically, scaffolds fail to fully reproduce the native organization of the microvascular tree in tissue-engineered grafts (Vajda et al., 2021). This problem is further heightened when such attempts are made using partial networks within complex tissue-engineered scaffolds (Corridon, 2021; Corridon, 2022).

Another area that greatly concerns the tissue engineering and regenerative medicine communities is the inability to construct vascular grafts that mimic native vessels. Such grafts must be resistant to thrombosis and infection, sensitive to vasoactive mediators, show the capacity for somatic growth, and possess long-term patency that supports ideal contractile and relaxative properties (Dimitrievska and Niklason, 2018). The shortage of transplantable vascular grafts is a rising concern as there is an increase in patients with cardiovascular conditions worldwide. Research has focused on various techniques to induce angiogenesis in scaffolds to address this imbalance. For instance, recently, Hancock et al. showed that precision micropuncture of the recipient’s vasculature could induce collagen angiogenesis and significantly increase physiologic perfusion and capillary network formation (Hancock et al., 2021). In addition, various synthetic blood vessels have been created by employing polymers, stem cells, and autologous grafting (Wilhelmi et al., 2014; Wang et al., 2021); however, conventional issues arising from the inherent thrombogenicity of synthetic materials persist (Greisler, 1990; Leal et al., 2021). They subsequently fail under different circumstances related with their non-regenerative properties, causing chronic inflammatory responses, ultimately affecting the graft’s structural integrity and function (Baguneid et al., 2006; Durán-Rey et al., 2021; Naegeli et al., 2022), resulting in excessive hyperplasia (Ao et al., 2000; Jeong et al., 2020), which altogether continue to limit clinical translation.

On the contrary, tissue-engineered vascular grafts (TEVGs) can integrate, remodel, grow, and repair the vascular wall upon implantation and provide suitable environments for tissue formation. Their close resemblance to native vessels provides a far more hemodynamic-responsive conduit that may better maintain patency, evade immune responses, and adapt to *in vivo* vascular dynamics (Matsuzaki et al., 2019). Nevertheless, the multifaceted nature of TEVGs makes them challenging to design with apt blood–material interactions (Ocak et al., 2013; Thamer et al., 2018). Also, from a commercial perspective, there is a dire need to devise ways to improve cell harvesting and production procedures, enhanced long-term storage, and reduce production times and costs.

This article draws attention to several significant barriers in VTE, which include the lack of functional vasculature, somatic growth, thrombosis, immune rejection, and mechanical failure, and highlights what is being done to overcome them. We believe developing vascular segments that mimic native vessels and address the previously mentioned challenges would substantially contribute to the field. An in-depth understanding of cell-surface interactions, hemodynamic forces, and surface topography is needed to design TEVGs with higher patency. Even with the advances in tissue engineering and regenerative medicine, the cellular response is still far from mimicking the biological function of native vessels, and the understanding of the interactions between blood cells and the vascular wall with the biomaterial post-transplantation is much needed. These interactions tend to change over time after implantation, and the cascade of events at every phase needs to be well understood. During the first few hours, a foreign body response and inflammatory and hemostatic processes govern the interactions between the TEVG, the cells, and the blood components at the implantation site. After the first few months, this phenomenon changes, and the adaptation to the hemodynamic behavior at the implantation site, dominates the TEVG integration. Lastly, the TEVG starts to perform its intended function and remodels tissue in response to the governing hemodynamics and cell turnover. If patency loss occurs within the first days or weeks, it is usually induced by thrombosis. Thereafter, this event may lead to hyperplasia intima in which the mechanical properties of the vascular wall are inadequate to support the blood flow pressures (Wautier and Wautier, 2013; Vahidkhah et al., 2014). In the long-term, atherosclerosis might occur, in which a chronic inflammatory process modifies the local calcium metabolism, causing stenosis and hardening the vascular wall, leading to their failures (Radke et al., 2018; Saito et al., 2021).

### 1.1 Lack of functional vasculature

Engineered scaffolds that are substitutes for native tissues/organs have failed repeatedly due to a lack of functional vasculature. So far, the gold standard for inducing angiogenesis in scaffolds relies on incorporating essential bioactive molecules within the scaffolds and providing their temporal release (Turner et al., 2017; Kuttappan et al., 2018; Ruskowitz and DeForest, 2018; Wang D. et al., 2019; Stejskalova et al., 2019; Mastrullo et al., 2020; Li et al., 2021; Shokrani et al., 2022). Pathophysiological stimuli can hinder scaffold performance in many cases as opposed to simple diffusion and degradation-based release, which occur typically. Such stimuli are generated from ischemia, inflammation, and elevated mechanical stress arising from unregulated cell growth with scaffolds that block the release of bioactive molecules. However, under normal conditions, where localized angiogenesis is permitted, various vital factors still limit their applications on a large scale. Specifically, these biomolecules have a short half-life and rapidly degrade *in vivo* (Mastrullo et al., 2020). Thus, their function within the scaffolds is complicated by natural pharmacokinetic and degradative processes that can occur post-transplantation.

Cell patterning is a new trend being followed extensively that directly offers a spatial control of angiogenesis, closely emulating the natural phenomenon (Akintewe et al., 2017; Yuan et al., 2022). 3D bioprinting (Zhang et al., 2021) and electrospinning (Jin et al., 2022) are the key players in cell patterning, wherein cells, biomaterials, and bioactive molecules are merged in bio-inks or electrically woven into complex designs with controlled pore sizes, microscopic channels, and nanofiber-based vascular networks. Bioprinting offers innovative solutions to shortcomings of traditional scaffolds by creating customizable vascular segments and networks within the engineered tissue constructs (Jia et al., 2016; Sarker et al., 2018; Fazal et al., 2021). In comparison, electrospinning provides ways to accurately control nanofiber diameter, alignment, degradation rate, and porosity (Islam et al., 2019). Additionally, it has been recently reported that electrospun nanofibers have diameters similar to natural ECM and thus replicate natural topographical cues that facilitate vessel growth (Kenar et al., 2019), integration into host vasculature (Cui et al., 2017), and angiogenesis (Gao et al., 2017). Most importantly, the stress exerted on cells during extrusion in 3D printing and electrospinning can be fatal and lead to their inhomogeneous distribution across scaffolds. As a result, further studies are needed to ensure their clinical applications.

### 1.2 Somatic growth of the artificial grafts

Since the first tissue-engineered blood vessel was developed in 1986 (Weinberg and Bell, 1986; Song et al., 2018; Naegeli et al., 2022) until today, several techniques, biomaterials, and cell sources have been employed to create replacement vessels. Even though we have made substantial advancements in this area, we still lack ways to comprehensively recreate human blood vessels’ structural and functional capacities (Hoenig et al., 2005). Simultaneously, the number of people suffering from arterial diseases and disorders has risen significantly. As a result, current treatment options remain limited to vascular bypass graft procedures (Cui et al., 2010; Vartanian and Conte, 2015; Li et al., 2022), and there is a definite need for alternatives.

One major hurdle synthetic scaffolds must overcome is somatic growth, particularly for pediatric and adolescent patients in prime growth phases. Significant efforts were made by Shin’oka et al. (2001) (Matsumura and Shinoka, 2015) and Hoerstrup et al. (2006) toward developing bioartificial vessels. This group seeded autologous cells on synthetic biodegradable polymer tubes, resulting in graft growth and remodeling, and this study has progressed to clinical trials. Nevertheless, specialized tasks are still needed to harness autologous cells from patients and then expand them to grow on synthetic scaffolds, thus making this problematic for routine clinical practices.

In another study, Syedian et al. (2016) reported exciting results from their efforts to develop off-the-shelf vascular grafts with native-like physiological strengths and stiffnesses. These grafts were crafted from a fibrin glue and fashioned into tubes remodeled by fibroblasts and then decellularized to generate acellular bioartificial grafts. Such tubular structures were implanted in lambs and evaluated when they reached adulthood. The implanted grafts displayed extensive recellularization, which consisted of complete luminal endothelialization, extensive elastin and collagen deposition, and signs of extraordinary somatic growth. After further standardizations and clinical trials, these vascular conduits may serve as permanent bioartificial grafts, specifically for pediatric and adolescent patients. However, it will be interesting to see how processes can evolve to simplify the cell culture and tubular formation processes and enhance this method’s utility.

It is no surprise that the inability of the graft to keep up with the surrounding tissue growth rate results in biomechanical properties incompatible with native vessels. This issue, in turn, causes neointima formation leading to immune system activation and resulting in graft failure (Mallis et al., 2020). In a similar work, Li et al. designed braided tube-reinforced poly (l-lactic acid-co-ε-caprolactone)/silk fibroin small-diameter vascular grafts and tested them in the *in vitro* biomimetic environment (Li and Zhao, 2019). The results were impressive, where no apparent degradation was observed. However, there were some changes in the grafts’ mechanical properties. Additionally, the study presented short-term degradation data, which is insufficient to draw factual inferences on their viability. Again, these issues would be compounded in more complex structures, as the absence of adequate vascularization would limit growth and support necrosis (Lebedenko and Banerjee, 2021). Nevertheless, preclinical and clinical studies have demonstrated vascular conduit biological growth capacity.

### 1.3 Targeting *in vivo* thrombosis

Thrombosis is an important defense mechanism to combat bleeding, but it also imposes a meaningful obstruction to developing tissue-engineered grafts. Thrombogenicity is also a critical factor in determining the biocompatibility of vascular grafts and the viability of vascular trees within complex organs (Jouda et al., 2022). For instance, the effects of thrombosis can be amplified when various synthetic grafts are used as vascular access devices for hemodialysis due to their low patency rates. The contemporary solution for hemodialysis involves creating an arteriovenous fistula, which is an abnormal connection between an artery and a vein and is considered an almost ideal model of vascular access (Stolic, 2013; Stoumpos et al., 2021). Nevertheless, these access lines constantly fail due to on-site clotting, hyperplasia, or infection (Sarkar et al., 2007; Gilpin and Nichols, 2010; Dahl et al., 2011; Lawson et al., 2020). These ill effects again support the need for better alternatives. To this end, researchers have reported using autologous stem cells, fibroblasts, bone marrow, and endothelial cells for tissue-engineered vascular grafts. A study by Syedain et al. (2017) revealed successful recellularization, intact structural integrity, and the absence of immune rejection over 6 months. Promising results were also obtained after decellularized grafts were implanted into baboons and tested as hemodialysis access points. Such grafts may represent an additional surgical option for hemodialysis access. However, the complexities in designing these segments, their high production cost, and their long creation time are substantial translational impediments (Lin et al., 2018; Fazal et al., 2021).

Once again, another interesting study by Kirkton et al. (2019) produced bioartificial acellular blood vessels for use as hemodialysis access lines. In that study, the authors performed preclinical human studies on their grafts by seeding human vascular cells into biodegradable Polyglycolic acid-based tubular scaffolds. Over time, the cells produce their own ECM, and the polymer is degraded. The scaffolds were then decellularized to leave behind a collagen-based matrix that retained sufficient biomechanical properties even after storage in phosphate-buffered saline solution for a year. Moreover, non-inflammatory host progenitor and vascular cells were incorporated into the grafts after implantation. These results provided evidence of a substantial *in vivo* transition that allowed these vascular segments to display a self-healing capacity after cannulation injury. It is the first-ever product to receive the Regenerative Medicine Advanced Therapy title from the U.S. Food and Drug Administration (Hayat et al., 2019; Kirkton et al., 2019). This designation allows accelerated approval of medical technologies intended to treat severe or life-threatening conditions.

Anticipated steps toward introducing artificial intelligence (AI) approaches to support automated production may further enhance this technology’s clinical potential, help mitigate challenges, and expand the future of VTE (Xu et al., 2005; Nguyen et al., 2019; Krackov et al., 2021). AI approaches have expanded our fundamental understanding of biological processes and are set to deliver enhanced medical solutions at the population and personal levels (Corridon et al., 2022). Within this context, AI is poised to become an invaluable tool to improve patient outcomes. Specifically, VTE strategies will benefit from AI-driven machine-optimized polymer synthesis, predictive modeling of scaffold fabrication processes, complex analyses of structure-function relationships, and deep learning of spatialized cell phenotypes and tissue composition (Guo et al., 2022). Altogether, the future of vascular engineering will benefit from improved biomaterial configuration, cell and tissue modeling, and scaffold fabrication.

### 1.4 Mechanics of the blood vessel walls and autoregulation

Autoregulation is a well-known phenomenon that allows vascular compartments to adapt to pressure changes to maintain blood flow. However, this physiological phenomenon exists in the innate vasculature and is absent in TEVGs (Corridon, 2021; Wang et al., 2022a). VTE has advanced to develop grafts that are strong enough to withstand hemodynamic forces, but the limitation still lies in their ability to autoregulate blood flow. The native blood vessels are a prime example of this law and form the basis of many pathological and physiological phenomena within the circulatory system (Pontiga and Gaytán, 2005). For instance, the enormous influence that vessel diameter has on the blood flow rate that circulates through the vessel is described by Poiseuille’s law. This law, $Q=\frac{∆P\pi r^{4}}{8\ln}$ , demonstrates how blood flow (Q) can be modeled as a function of blood pressure (P), viscosity (η), vessel length (L), and radius (r). From this equation, flow rate is directly proportional to the pressure gradient and the radius is raised to the fourth power. This relationship can be used to illustrate the effect of arteriosclerotic plaque accumulation. During arteriosclerosis, plaque build-up in the vascular lumen can reduce the vessel radius and dramatically increase blood pressure, which has been known to contribute to irreparable damage to the vascular wall.

The multilayered structural complexity of arterial blood vessels helps them maintain tissue homeostasis by regulating their biological and physical properties. Blood vessels show viscoelastic properties, undergoing creep and relaxation when placed under load. The elastic properties are due to the presence of elastin, while collagen and smooth muscle cells contribute to the viscous behavior (Akentjew et al., 2019). Thus, their intrinsic ability to dilate and constrict in response to dynamic perfusion pressure changes maintains constant blood flow and avoids occlusion or failures. The large arterial vessels act as capacitors and maintain pressure by altering resistance mediated *via* physiological feedback. The three homeostatic mechanisms, neural, endocrine, and autoregulatoration, ensure adequate blood pressure, blood flow, distribution, and perfusion. Blood vessels contain baroreceptors that continuously monitor blood pressure and cause vasoconstriction or vasodilation as needed (Chopra et al., 2011). Therefore, endothelial cells, smooth muscle cells, fibroblast, collagen, and elastin synergistically control physiological functions of the blood vessels, such as vasoconstriction, vasodilatation and extracellular matrix production, and pathogenesis of vascular diseases (Lucie et al., 2018).

Introducing autoregulative adaptations, which are inherent to native vessels, into bioartificial grafts has been a goal of tissue engineering and has still not been realized. The inability of these grafts to adapt the blood pressure leads to intimal hyperplasia, changes in blood viscosity, and stenosis, all contributing towards thrombosis. The failure of TEVGs to adequately adjust to hemodynamic forces and changes in blood pressure causes the graft to narrow, that inadvertently increases the pressure, similar to arteriosclerosis, and ultimately results in their failure. An in-depth understanding of the cell-substrate interface has opened new biomimetic approaches to manipulate and regulate vascular cell functions in bioartificial grafts. Various methods, such as growing longitudinally aligned endothelial cells (Avci-Adali et al., 2010; Nakayama et al., 2016) and circumferentially aligned SMSCs (Cao et al., 2010; Chen et al., 2020), on graft surface, multilayered vascular grafts, and nanolamellar lumen topography (Wang Z. et al., 2019) have been developed to mimic the native-like ECM, cellular, and structural arrangement in TEVGs.

Mechanical strength and adaptation to hemodynamic changes are needed to successfully design and implant TEVGs. The combination of geometric resemblance, dedicated endothelialization, and alignment of vascular cells are indispensable to designing grafts that mimic native structures. This arrangement can positively affect SMC phenotype, increase M2 macrophage infiltration, and enhance extracellular matrix formation, thereby promoting proper cellular functions. Computational approaches have offered interesting methods to design structures with suitable topography, precise flexibility, and adequate mechanical strength. They have proven to match better a target vessel’s compliance, diameter, and wall thickness by computationally tuning its layered composition (Furdella et al., 2021). Ideally, more importance should be given to grafts that can be adjusted for the biomechanical response, withstand hemodynamic blood pressures, and offer efficient remodeling over time.

### 1.5 Immune responses

While several novel technologies and designs have come up serving as grafts for damaged vessels, a very crucial aspect leading to their failure still needs to be noticed- the immune system. Graft rejection is caused by the immune system identifying the transplant as foreign, triggering a response that ultimately destroys the transplanted organ or tissue. Foreign bodies are presented to the immune system in the form of small molecules called antigens, and identifying these non-self antigens triggers an immune response. Reports have suggested that a major factor for patency loss and the subsequent failure of vascular grafts is the development of pro-coagulant and inflammatory phenotypes of the interacting blood cells and the cells in the vascular wall near the implantation site (Li et al., 2010; Gaudino et al., 2017). In addition, the post-implantation microenvironment presents an altered physiology, activated signaling cascades from the immune system, increased coagulation proteins, platelet adhesion and activation, and new hemodynamics (Rodriguez-Soto et al., 2021). Consequently, failure of the grafts starts immediately after implantation due to foreign body reaction and platelet aggregation-driven thrombus formation or in later phases owing to the interactions of the graft with immune cells and the absence of hemodynamic stabilization.

Besides being the gatekeeper, the host immune response also plays a significant role in guiding TEVG remodeling and regulating stenosis (Roh et al., 2010; Hibino et al., 2015). While it remains unknown how specific mechanisms of immune cells regulate graft remodeling, macrophages have been pointed out as key players (Graney et al., 2020). The early recruitment of these innate immune cells at the graft surface coordinates the subsequent level of tissue regeneration. The recruited cells then respond to substrates and create a microenvironment by releasing a series of cytokines and chemokines that regulate the subsequent tissue regeneration or directly participate in the regeneration process. A low level of early cell recruitment impedes tissue cell recruitment and proliferation. In contrast, excessive cell recruitment can lead to early narrowing or stenosis of the vascular graft and rapid scaffold remodeling (Hibino et al., 2011a; Hibino et al., 2011b; Matsuzaki et al., 2021).

A delicate balance between the M1 pro-inflammatory and M2 anti-inflammatory responses is needed to regulate the remodeling cascade effectively. Studies have shown that biomaterial physicochemical properties (Padmanabhan et al., 2014; Wang et al., 2014; Liu et al., 2019; Sridharan et al., 2019; Wissing et al., 2019; Furdella et al., 2021), their chemical composition (Sadtler et al., 2019; Furdella et al., 2021), and the choice of cells play a crucial role in macrophage responses. Opposite to suppressing the immune system or centering on bio-inertness, research is needed to focus on harnessing the immune system’s role in the remodeling process and trying to direct immune cells to set up a pro-regeneration microenvironment to promote tissue remodeling in a preferred direction. A future direction should be to synergize tissue and immune engineering aspects. Instead of avoiding the detection by the immune system, recent research has started to appreciate the role of the immune system in the remodeling process and tried to direct immune cells to set up a pro-regeneration microenvironment to promote tissue remodeling in a preferred direction. Additionally, computational approaches that combine surface topography, structural parameters, and hemodynamics are being used to improve the current understanding of the underlying mechanisms of blood-biomaterial interactions (Riveros et al., 2022). Multiparametric methods used in this manner can support the study of protein adsorption on TEVGs, which can be extended to analyze the effect of hemodynamic variables over the adsorption of plasma proteins to improve endothelialization and evade immune responses.

## 2 Clinical applications and future perspective

Advancements in technologies in recent decades have marked colossal progress in developing translatable TEVGs, with few technologies already reaching advanced stages of human clinical trials. Shin’oka’s group performed the first reported clinical application of a TEVG in 2001 using a biodegradable pulmonary conduit made of peripheral vein derivative of primary Vascular Smooth Muscle Cells from the same individual and PGA-reinforced scaffold, implanted in a child with congenital pulmonary atresia (Shin’oka et al., 2001). With patency being maintained for 7 months post-implantation, this study was expanded to 23 more pediatric patients with the same condition (NCT01034007) (https://clinicaltrials.gov/ct2/show/NCT01034007), giving no graft patency even after 5.8 years (Shin’oka, 2004). Recently, PGA and PCLA (Polycaprolactone-co-lactide) copolymer scaffolds were seeded with bone marrow mononuclear cells seeded were approved for clinical trials. These second-generation scaffold trials (NCT04467671) (https://clinicaltrials.gov/ct2/show/NCT04467671) have been active since July 2020 and claim to give better results than previous projects. Further, L’Heureux introduced the Lifeline graft composed of ECM produced by culturing and maturation of autologous cells (L’Heureux et al., 2006). The graft offers several advantages in terms of its mechanical strength, excellent integration with surrounding tissue, thrombotic resistance, and eliminating the need for exogenous scaffolding (Peck et al., 2012).

Unfortunately, of the nine patients who received a graft (NCT00850252) (https://clinicaltrials.gov/ct2/show/NCT00850252), three of these grafts failed from thrombosis or rejection after 6 months (McAllister et al., 2009). Even though the graft successfully mimicked the burst pressure of the human internal mammary artery, a six to 9-month production timeline with high costs limits the mass-scale applicability of the grafts to be used in routine clinical practices. However, this sparked interest in using decellularized ECM-based scaffolds for engineered vessels using which Lawson et al. developed Humacyte grafts for end-stage renal (ESR) failure patients, providing arterio-venous shunt access for hemodialysis (NCT01744418 and NCT01840956) (https://clinicaltrials.gov/ct2/show/NCT01744418 and https://clinicaltrials.gov/ct2/show/NCT01840956) (Lawson et al., 2016). ESR patients experience vascular damage due to repeated needle punctures for hemodialysis access, and available synthetic expanded Polytetrafluoroethylene (ePTFE) grafts and arterio-venous fistula vascular access options impose high infection rates and lack of self-healing (Naegeli et al., 2022). Results impressively showed 63% patency at 6 months but drastically reduced to only 18% at 18 months. However, the Humacyte grafts have entered a new Phase 2 clinical trial (NCT03005418) exploring the possibility of treating damaged vessels after vascular trauma (https://clinicaltrials.gov/ct2/show/NCT03005418). However, complex design and high cost of production still stand as significant hurdles. The focus needs to be mainly on simplifying the design and optimizing manufacturing processes to create off-the-shelf TEVGs for patients in urgent need of the product. Dahl et al. have taken an appealing step in this direction (Dahl et al., 2003), but it would still need time and effort to bring the product into routine clinical practice. Leading groups worldwide, such as Xeltis and Humacyte, Inc., have developed enticing alternatives for venous and arterial flow systems to advance patient care; however, their TEVG has yet to hit the market.

Graft patency, efficacy, site infection, inflammation, acute and chronic adverse events, graft length and volume, cell infiltration and viability, stenosis, and mechanical stability are a few parameters that should be comprehensively tested as primary outcome measures to ensure their long-term viability. To summarize, the grafts under clinical trials showed impressive results for a shorter duration. However, the majority failed in giving satisfactory performance in the long-term. A summary of a few promising ongoing clinical trials for TEVGs is presented in Table 1.

**TABLE 1:** Ongoing clinical trials for TEVGs (Home).

Title	Identifier	Sample size	Status/phase	Primary/secondary endpoints	Sponsor
Two-year study of the safety and efficacy of the second-generation tissue engineered vascular grafts (TEVG-2)	NCT04467671	24 participants	Currently in phase II	The primary outcome will be measured in terms of safety and tolerability. Secondary measures will be efficacy based on graft volume and graft length. Patients are being monitored for adverse and serious adverse events, especially stenosis.	Nationwide Children’s Hospital
Evaluation of the safety and efficacy of a vascular prosthesis as an above-knee bypass graft in patients with PAD	NCT01872208	20 participants	Active, not recruiting	To assess the safety and efficacy of a novel human acellular vessel to be used as above-knee femoro-popliteal bypass graft in patients with peripheral arterial disease. Primary outcome measures will be based on aneurysm formation, anastomotic bleeding or rupture, graft infection and irritation/inflammation/infection at the implantation in a time frame of 5 days–24 months.	Humacyte, Inc.
Evaluation of the safety and efficacy of a vascular prosthesis for hemodialysis access in patients with ESRD	NCT01744418	40 participants	Active, not recruiting	To assess the safety and efficacy of a novel human acellular vascular graft intended as an alternative to synthetic materials and to autologous grafts in the creation of vascular access for dialysis. Graft patency, safety and tolerability for 6 months are the primary outcome measures.	Humacyte, Inc.
Humacyte human acellular vessel (HAV) in patients with vascular trauma	NCT03005418	100 participants	Currently in phase II	This study evaluates the use of the Human Acellular Vessel (HAV) in adults with vascular trauma below the neck who are undergoing vascular reconstructive surgery. Primary outcome measures are patency for a time frame of 30 days and severity of adverse events for 36 months.	Humacyte, Inc.
Humacyte’s HAV for femoro-popliteal bypass in patients With PAD	NCT02887859	15 participants	Currently in phase II	This study will evaluate the patency and safety of humacyte’s human acellular vessel when surgically implanted into a leg to improve blood flow in patients with peripheral arterial disease. Patency, aneurysm formation, anastomotic bleeding or rupture, infection, and severity of adverse events are primary measures for a time frame of 12 months.	Humacyte, Inc.
Safety and efficacy assessment of HAV (manufactured using large-scale system) in patients needing vascular access for dialysis	NCT04135417	30 participants	Currently in phase II	Safety and efficacy assessment of surgically implanted HAV to be used as vascular conduit for hemodialysis vascular access. Adverse events, graft patency, and antibodies production are primary outcome measures for a time frame of 2–3 months post transplantation.	Humacyte, Inc.
Comparison of the human acellular vessel (HAV) with fistulas as conduits for hemodialysis	NCT03183245	240 participants	Currently in phase III	Comparison of the Human Acellular Vessel with arteriovenous fistula when used for hemodialysis access. The patients will be assessed for functional and secondary patency at 6 and 12 months respectively. Other secondary outcome measures will be histopathological remodeling, site infections, adverse events, and long-term patency.	Humacyte, Inc.
Xeltis coronary artery bypass graft (XABG) first in human (FIH) (XABG-FIH)	NCT04545112	15 participants	Enrolling by invitation	Assessing preliminary device safety, feasibility, and performance data of the XABG in patients. Primary outcome will be measured in terms of procedural success in first 30 days and no severe adverse events. Secondary measures will be graft patency, no intimal hyperplasia, no infection, and lumen diameter uniformity.	Xeltis
Vascular graft infections (VASGRA)	NCT01821664	1,800 participants	Recruiting	The clinical trial looks into the epidemiology, best treatment options, imaging modalities, and impact of negative pressure wound therapy. Graft infection within 10 years is the primary outcome measure, while bleeding, foreign body reactions and all cause-mortality serve as secondary endpoints for the same time frame.	University of Zurich

From a translational viewpoint, ideal vascular grafts must possess the following vital properties: 1) Bio-inertness, 2) cost-effectiveness, 3) “off-the-shelf” availability, 4) easy handling and storage, 5) scalability, 6) resistance to stenosis, thrombosis, and infection, 7) ability to support somatic growth, and 8) mechanically robust to sustain blood pressure without subsequent aneurysmal dilatation. With the growing technological advancements, researchers are working towards developing robust computational and artificial intelligence models that can effectively predict scaffold properties and *in vivo* performance with high accuracy, giving reliable vascular grafts. Establishing an intricate balance between graft degradation rate and neo-tissue formation is another challenging aspect of successful graft remodeling. The dynamics between blood flow, protein adsorption, mechanical stimuli, and cell adhesion are essential to understanding the outcomes of tissue-engineered vascular grafts (TEVGs). TEVG remodeling heavily relies on the recruitment of host cells. Therefore, an in-depth study on which cells populate the graft after implantation and their mechanisms, focusing on fallout and transmural cell growth, must be validated before implementing arterial TEVGs in humans.

In conclusion, there are several key hurdles that current vascular grafting methods must overcome. Cell-free bioresorbable synthetic TEVGs, till now, seem to be the most suitable economic option for translation; however, their “off-the-shelf” availability still requires rigorous efforts. Furthermore, the immune system’s major obstacle must be checked, and immunomodulation approaches must be identified to achieve efficient graft remodeling. Moreover, a better understanding of the synergistic effect of graft design, cellular cascades, and the subsequent mechanical and immunological responses will provide a means to control healing and remodeling.

## References

[B1] Akentjew T. L. Terraza C. Suazo C. Maksimcuka J. Wilkens C. A. Vargas F. (2019). Author Correction: Rapid fabrication of reinforced and cell-laden vascular grafts structurally inspired by human coronary arteries. Nat. Commun. 10 (1), 3508. 10.1038/s41467-019-11446-9 31366976 PMC6668386

[B2] Akintewe O. O. Roberts E. G. Rim N-G. Ferguson M. A. H. Wong J. Y. (2017). Design approaches to myocardial and vascular tissue engineering. Annu. Rev. Biomed. Eng. 19 (1), 389–414. 10.1146/annurev-bioeng-071516-044641 28471698

[B3] Ao P. Y. Hawthorne W. J. Vicaretti M. Fletcher J. P. (2000). Development of intimal hyperplasia in six different vascular prostheses. Eur. J. Vasc. endovascular Surg. 20 (3), 241–249. the official journal of the European Society for Vascular Surgery. 10.1053/ejvs.2000.1177 10986022

[B4] Avci-Adali M. Ziemer G. Wendel H. P. (2010). Induction of epc homing on biofunctionalized vascular grafts for rapid *in vivo* self-endothelialization — a review of current strategies. Biotechnol. Adv. 28 (1), 119–129. 10.1016/j.biotechadv.2009.10.005 19879347

[B5] Baguneid M. S. Seifalian A. M. Salacinski H. J. Murray D. Hamilton G. Walker M. G. (2006). Tissue engineering of blood vessels. Br. J. Surg. 93 (3), 282–290. 10.1002/bjs.5256 16498591

[B6] Blume C. Kraus X. Heene S. Loewner S. Stanislawski N. Cholewa F. (2022). Vascular implants - new aspects for *in situ* tissue engineering. Eng. Life Sci. 22 (3-4), 344–360. 10.1002/elsc.202100100 35382534 PMC8961049

[B7] Cao Y. Poon Y. F. Feng J. Rayatpisheh S. Chan V. Chan-Park M. B. (2010). Regulating orientation and phenotype of primary vascular smooth muscle cells by biodegradable films patterned with arrays of microchannels and discontinuous microwalls. Biomaterials 31 (24), 6228–6238. 10.1016/j.biomaterials.2010.04.059 20537704

[B8] Chen M. Li L. Xia L. Zhang F. Jiang S. Hu H. (2020). Temperature responsive shape-memory scaffolds with circumferentially aligned nanofibers for guiding smooth muscle cell behavior. Macromol. Biosci. 20 (2), 1900312. 10.1002/mabi.201900312 31854123

[B9] Chopra S. Baby C. Jacob J. J. (2011). Neuro-endocrine regulation of blood pressure. Indian J. Endocrinol. metabolism 15 (4), S281–S288. 10.4103/2230-8210.86860 PMC3230096 22145130

[B10] Corridon P. R. (2021). *In vitro* investigation of the impact of pulsatile blood flow on the vascular architecture of decellularized porcine kidneys. Sci. Rep. 11 (1), 16965. 10.1038/s41598-021-95924-5 34417499 PMC8379263

[B11] Corridon P. R. (2022). Intravital microscopy datasets examining key nephron segments of transplanted decellularized kidneys. Sci. Data 9 (1), 561. figshare. 10.1038/s41597-022-01685-9 36088356 PMC9464233

[B12] Corridon P. R. Ko I. K. Yoo J. J. Atala A. (2017). Bioartificial kidneys. Curr. Stem Cell Rep. 3 (2), 68–76. 10.1007/s40778-017-0079-3 33005562 PMC7526744

[B13] Corridon P. R. Rhodes G. J. Leonard E. C. Basile D. P. Gattone V. H. 2nd Bacallao R. L. (2013). A method to facilitate and monitor expression of exogenous genes in the rat kidney using plasmid and viral vectors. Am. J. Physiol. Ren. Physiol. 304 (9), F1217–F1229. 10.1152/ajprenal.00070.2013 PMC3651629 23467422

[B14] Corridon P. R. Wang X. Shakeel A. Chan V. (2022). Digital technologies: Advancing individualized treatments through gene and cell therapies, pharmacogenetics, and disease detection and diagnostics. Biomedicines 10 (10), 2445. 10.3390/biomedicines10102445 36289707 PMC9599083

[B15] Cui L. Li J. Long Y. Hu M. Li J. Lei Z. (2017). Vascularization of LBL structured nanofibrous matrices with endothelial cells for tissue regeneration. RSC Adv. 7 (19), 11462–11477. 10.1039/c6ra26931a

[B16] Cui Y. C. Hao X. H. Huang F. J. Li J. H. Lai Y. Q. Zhou Q. W. (2010). Coronary artery bypass grafting in adults with congenital heart disease. J. cardiac Surg. 25 (6), 629–632. 10.1111/j.1540-8191.2010.01110.x 21029160

[B17] Dahl S. L. Koh J. Prabhakar V. Niklason L. E. (2003). Decellularized native and engineered arterial scaffolds for transplantation. Cell Transplant. 12 (6), 659–666. 10.3727/000000003108747136 14579934

[B18] Dahl S. L. Kypson A. P. Lawson J. H. Blum J. L. Strader J. T. Li Y. (2011). Readily available tissue-engineered vascular grafts. Sci. Transl. Med. 3 (68), 68ra9. 10.1126/scitranslmed.3001426 21289273

[B19] Devillard C. D. Marquette C. A. (2021). Vascular tissue engineering: Challenges and requirements for an ideal large scale blood vessel. Front. Bioeng. Biotechnol. 9, 721843. 10.3389/fbioe.2021.721843 34671597 PMC8522984

[B20] Dimitrievska S. Niklason L. E. (2018). Historical perspective and future direction of blood vessel developments. Cold Spring Harb. Perspect. Med. 8 (2), a025742. 10.1101/cshperspect.a025742 28348177 PMC5685928

[B21] Durán-Rey D. Crisóstomo V. Sánchez-Margallo J. A. Sánchez-Margallo F. M. (2021). Systematic review of tissue-engineered vascular grafts. Front. Bioeng. Biotechnol. 9, 771400. 10.3389/fbioe.2021.771400 34805124 PMC8595218

[B22] Fazal F. Raghav S. Callanan A. Koutsos V. Radacsi N. (2021). Recent advancements in the bioprinting of vascular grafts. Biofabrication 13 (3), 032003. 10.1088/1758-5090/ac0963 34102613

[B23] Furdella K. J. Higuchi S. Behrangzade A. Kim K. Wagner W. R. Vande Geest J. P. (2021). *In-vivo* assessment of a tissue engineered vascular graft computationally optimized for target vessel compliance. Acta biomater. 123, 298–311. 10.1016/j.actbio.2020.12.058 33482362 PMC7920940

[B24] Gao W. Jin W. Li Y. Wan L. Wang C. Lin C. (2017). A highly bioactive bone extracellular matrix-biomimetic nanofibrous system with rapid angiogenesis promotes diabetic wound healing. J. Mater. Chem. B 5 (35), 7285–7296. 10.1039/c7tb01484h 32264178

[B25] Gaudino M. Antoniades C. Benedetto U. Deb S. Di Franco A. Di Giammarco G. (2017). Mechanisms, consequences, and prevention of coronary graft failure. Circulation 136 (18), 1749–1764. 10.1161/CIRCULATIONAHA.117.027597 29084780

[B26] Gilpin V. Nichols W. K. (2010). Vascular access for hemodialysis: Thrills and thrombosis. J. Vasc. Nurs. 28 (2), 78–83. official publication of the Society for Peripheral Vascular Nursing. 10.1016/j.jvn.2010.03.001 20494299

[B27] Graney P. L. Ben-Shaul S. Landau S. Bajpai A. Singh B. Eager J. (2020). Macrophages of diverse phenotypes drive vascularization of engineered tissues. Sci. Adv. 6 (18), eaay6391. 10.1126/sciadv.aay6391 32494664 PMC7195167

[B28] Greisler H. P. (1990). Interactions at the blood/material interface. Ann. Vasc. Surg. 4 (1), 98–103. 10.1007/BF02042699 2297480

[B29] Guo J. L. Januszyk M. Longaker M. T. (2022). Machine learning in tissue engineering. Tissue Eng. Part A. 10.1089/ten.TEA.2022.0128 PMC9885550 35943870

[B30] Hancock P. C. Koduru S. V. Sun M. Ravnic D. J. (2021). Induction of scaffold angiogenesis by recipient vasculature precision micropuncture. Microvasc. Res. 134, 104121. 10.1016/j.mvr.2020.104121 33309646

[B31] Hayat S. M. G. Farahani N. Safdarian E. Roointan A. Sahebkar A. (2019). Gene delivery using lipoplexes and polyplexes: Principles, limitations and solutions. Crit. Rev. Eukaryot. Gene Expr. 29 (1), 29–36. 10.1615/CritRevEukaryotGeneExpr.2018025132 31002592

[B32] Hibino N. Mejias D. Pietris N. Dean E. Yi T. Best C. (2015). The innate immune system contributes to tissue-engineered vascular graft performance. FASEB J. 29 (6), 2431–2438. official publication of the Federation of American Societies for Experimental Biology. 10.1096/fj.14-268334 25713026 PMC4447224

[B33] Hibino N. Villalona G. Pietris N. Duncan D. R. Schoffner A. Roh J. D. (2011). Tissue-engineered vascular grafts form neovessels that arise from regeneration of the adjacent blood vessel. FASEB J. 25 (8), 2731–2739. official publication of the Federation of American Societies for Experimental Biology. 10.1096/fj.11-182246 21566209 PMC3136337

[B34] Hibino N. Yi T. Duncan D. R. Rathore A. Dean E. Naito Y. (2011). A critical role for macrophages in neovessel formation and the development of stenosis in tissue-engineered vascular grafts. FASEB J. 25 (12), 4253–4263. official publication of the Federation of American Societies for Experimental Biology. 10.1096/fj.11-186585 21865316 PMC3236622

[B35] Hoenig M. R. Campbell G. R. Rolfe B. E. Campbell J. H. (2005). Tissue-engineered blood vessels: Alternative to autologous grafts? Arteriosclerosis, thrombosis, Vasc. Biol. 25 (6), 1128–1134. 10.1161/01.ATV.0000158996.03867.72 15705929

[B36] Hoerstrup S. P. Cummings Mrcs I. Lachat M. Schoen F. J. Jenni R. Leschka S. (2006). Functional growth in tissue-engineered living, vascular grafts: Follow-up at 100 weeks in a large animal model. Circulation 114 (1), I159–I166. 10.1161/CIRCULATIONAHA.105.001172 16820566

[B67] Home ClinicalTrials.gov [Internet]. Available at: https://clinicaltrials.gov/ (Accessed December 19, 2022).

[B37] Islam M. S. Ang B. C. Andriyana A. Afifi A. M. (2019). A review on fabrication of nanofibers via electrospinning and their applications. SN Appl. Sci. 1 (10), 1248. 10.1007/s42452-019-1288-4

[B38] Jeong Y. Yao Y. Yim E. K. F. (2020). Current understanding of intimal hyperplasia and effect of compliance in synthetic small diameter vascular grafts. Biomater. Sci. 8 (16), 4383–4395. 10.1039/d0bm00226g 32643723 PMC7452756

[B39] Jia W. Gungor-Ozkerim P. S. Zhang Y. S. Yue K. Zhu K. Liu W. (2016). Direct 3D bioprinting of perfusable vascular constructs using a blend bioink. Biomaterials 106, 58–68. 10.1016/j.biomaterials.2016.07.038 27552316 PMC5300870

[B40] Jin Q. Fu Y. Zhang G. Xu L. Jin G. Tang L. (2022). Nanofiber electrospinning combined with rotary bioprinting for fabricating small-diameter vessels with endothelium and smooth muscle. Compos. Part B Eng. 234, 109691. 10.1016/j.compositesb.2022.109691

[B41] Jouda H. Larrea Murillo L. Wang T. (2022). Current progress in vascular engineering and its clinical applications. Cells 11 (3), 493. 10.3390/cells11030493 35159302 PMC8834640

[B42] Kenar H. Ozdogan C. Y. Dumlu C. Doger E. Kose G. T. Hasirci V. (2019). Microfibrous scaffolds from poly(l-lactide-co-ε-caprolactone) blended with xeno-free collagen/hyaluronic acid for improvement of vascularization in tissue engineering applications. Mater. Sci. Eng. C, Mater. Biol. Appl. 97, 31–44. 10.1016/j.msec.2018.12.011 30678916

[B43] Kirkton R. D. Santiago-Maysonet M. Lawson J. H. Tente W. E. Dahl S. L. M. Niklason L. E. (2019). Bioengineered human acellular vessels recellularize and evolve into living blood vessels after human implantation. Sci. Transl. Med. 11 (485), eaau6934. 10.1126/scitranslmed.aau6934 30918113 PMC7557107

[B44] Krackov W. Sor M. Razdan R. Zheng H. Kotanko P. (2021). Artificial intelligence methods for rapid vascular access aneurysm classification in remote or in-person settings. Blood Purif. 50 (4-5), 636–641. 10.1159/000515642 33857941

[B45] Kuttappan S. Mathew D. Jo J. I. Tanaka R. Menon D. Ishimoto T. (2018). Dual release of growth factor from nanocomposite fibrous scaffold promotes vascularisation and bone regeneration in rat critical sized calvarial defect. Acta biomater. 78, 36–47. 10.1016/j.actbio.2018.07.050 30067947

[B46] L'Heureux N. Dusserre N. Konig G. Victor B. Keire P. Wight T. N. (2006). Human tissue-engineered blood vessels for adult arterial revascularization. Nat. Med. 12 (3), 361–365. 10.1038/nm1364 16491087 PMC1513140

[B47] Lawson J. H. Glickman M. H. Ilzecki M. Jakimowicz T. Jaroszynski A. Peden E. K. (2016). Bioengineered human acellular vessels for dialysis access in patients with end-stage renal disease: Two phase 2 single-arm trials. Lancet London, Engl. 387 (10032), 2026–2034. 10.1016/S0140-6736(16)00557-2 PMC4915925 27203778

[B48] Lawson J. H. Niklason L. E. Roy-Chaudhury P. (2020). Challenges and novel therapies for vascular access in haemodialysis. Nat. Rev. Nephrol. 16 (10), 586–602. 10.1038/s41581-020-0333-2 32839580 PMC8108319

[B49] Leal B. B. J. Wakabayashi N. Oyama K. Kamiya H. Braghirolli D. I. Pranke P. (2021). Vascular tissue engineering: Polymers and methodologies for small caliber vascular grafts. Front. Cardiovasc. Med. 7, 592361. 10.3389/fcvm.2020.592361 33585576 PMC7873993

[B50] Lebedenko C. G. Banerjee I. A. (2021). Enhancing kidney vasculature in tissue engineering-current trends and approaches: A review. Biomimetics (Basel) 6 (2), 40. 10.3390/biomimetics6020040 34208664 PMC8293130

[B51] Li S. Nih L. R. Bachman H. Fei P. Li Y. Nam E. (2017). Hydrogels with precisely controlled integrin activation dictate vascular patterning and permeability. Nat. Mater. 16 (9), 953–961. 10.1038/nmat4954 28783156 PMC5809173

[B52] Li X. Zhao H. (2019). Mechanical and degradation properties of small-diameter vascular grafts in an *in vitro* biomimetic environment. J. Biomater. Appl. 33 (8), 1017–1034. 10.1177/0885328218820751 30636493

[B53] Li Y. Liu Y. Bai H. Li R. Shang J. Zhu Z. (2021). Sustained release of VEGF to promote angiogenesis and osteointegration of three-dimensional printed biomimetic titanium alloy implants. Front. Bioeng. Biotechnol. 9, 757767. 10.3389/fbioe.2021.757767 34869265 PMC8634467

[B54] Li Z. Delaney M. K. O'Brien K. A. Du X. (2010). Signaling during platelet adhesion and activation. Arteriosclerosis, Thrombosis, Vasc. Biol. 30 (12), 2341–2349. 10.1161/ATVBAHA.110.207522 PMC3085271 21071698

[B55] Li Z. Qiao Y. Sheng W. Chi Y. (2022). Newly developed graft failure detected using computed tomography within 1 Year after coronary artery bypass grafting surgery: One single-center experience. Front. Cardiovasc. Med. 9, 779015. 10.3389/fcvm.2022.779015 35174230 PMC8841778

[B56] Lin C-H. Hsia K. Ma H. Lee H. Lu J-H. (2018). *In vivo* performance of decellularized vascular grafts: A review article. Int. J. Mol. Sci. 19 (7), 2101. 10.3390/ijms19072101 30029536 PMC6073319

[B57] Liu J. Qin Y. Wu Y. Sun Z. Li B. Jing H. (2019). The surrounding tissue contributes to smooth muscle cells' regeneration and vascularization of small diameter vascular grafts. Biomater. Sci. 7 (3), 914–925. 10.1039/c8bm01277f 30511718

[B58] Lovett M. Lee K. Edwards A. Kaplan D. L. (2009). Vascularization strategies for tissue engineering. Reviews 15 (3), 353–370. 10.1089/ten.TEB.2009.0085 PMC2817665 19496677

[B59] Lucie B. Martina T. Elena F. Roman M. Jana S. Jana M. (2018). The role of vascular smooth muscle cells in the physiology and pathophysiology of blood vessels. 10.5772/intechopen.77115

[B60] Mallis P. Kostakis A. Stavropoulos-Giokas C. Michalopoulos E. (2020). Future perspectives in small-diameter vascular graft engineering. Bioeng. (Basel) 7 (4), 160. 10.3390/bioengineering7040160 PMC7763104 33321830

[B61] Masson-Meyers D. S. Tayebi L. (2021). Vascularization strategies in tissue engineering approaches for soft tissue repair. J. Tissue Eng. Regen. Med. 15 (9), 747–762. 10.1002/term.3225 34058083 PMC8419139

[B62] Mastrullo V. Cathery W. Velliou E. Madeddu P. Campagnolo P. (2020). Angiogenesis in tissue engineering: As nature intended? Front. Bioeng. Biotechnol. 8, 188. 10.3389/fbioe.2020.00188 32266227 PMC7099606

[B63] Matsumura G. Shinoka T. (2015). First report of histological evaluation of human tissue-engineered vasculature. J. Biotechnol. Biomater. 5. 10.4172/2155-952x.1000200

[B64] Matsuzaki Y. John K. Shoji T. Shinoka T. (2019). The evolution of tissue engineered vascular graft technologies: From preclinical trials to advancing patient care. Appl. Sci. (Basel, Switz. 9 (7), 1274. 10.3390/app9071274 PMC6937136 31890320

[B65] Matsuzaki Y. Miyamoto S. Miyachi H. Iwaki R. Shoji T. Blum K. (2021). Improvement of a novel small-diameter tissue-engineered arterial graft with heparin conjugation. Ann. Thorac. Surg. 111 (4), 1234–1241. 10.1016/j.athoracsur.2020.06.112 32946845

[B66] McAllister T. N. Maruszewski M. Garrido S. A. Wystrychowski W. Dusserre N. Marini A. (2009). Effectiveness of haemodialysis access with an autologous tissue-engineered vascular graft: A multicentre cohort study. Lancet (London, Engl. 373 (9673), 1440–1446. 10.1016/S0140-6736(09)60248-8 19394535

[B68] Meng X. Xing Y. Li J. Deng C. Li Y. Ren X. (2021). Rebuilding the vascular network: *In vivo* and *in vitro* approaches. Front. Cell Dev. Biol. 9, 639299. 10.3389/fcell.2021.639299 33968926 PMC8097043

[B69] Mironov V. Kasyanov V. Markwald R. R. (2008). Nanotechnology in vascular tissue engineering: From nanoscaffolding towards rapid vessel biofabrication. Trends Biotechnol. 26 (6), 338–344. 10.1016/j.tibtech.2008.03.001 18423666

[B70] Naegeli K. M. Kural M. H. Li Y. Wang J. Hugentobler E. A. Niklason L. E. (2022). Bioengineering human tissues and the future of vascular replacement. Circ. Res. 131 (1), 109–126. 10.1161/CIRCRESAHA.121.319984 35737757 PMC9213087

[B71] Nakayama K. H. Surya V. N. Gole M. Walker T. W. Yang W. Lai E. S. (2016). Nanoscale patterning of extracellular matrix alters endothelial function under shear stress. Nano Lett. 16 (1), 410–419. 10.1021/acs.nanolett.5b04028 26670737 PMC4758680

[B72] Neishabouri A. Soltani Khaboushan A. Daghigh F. Kajbafzadeh A. M. Majidi Zolbin M. (2022). Decellularization in tissue engineering and regenerative medicine: Evaluation, modification, and application methods. Front. Bioeng. Biotechnol. 10, 805299. 10.3389/fbioe.2022.805299 35547166 PMC9081537

[B73] Nguyen A. H. Marsh P. Schmiess-Heine L. Burke P. J. Lee A. Lee J. (2019). Cardiac tissue engineering: State-of-the-art methods and outlook. J. Biol. Eng. 13 (1), 57. 10.1186/s13036-019-0185-0 31297148 PMC6599291

[B74] Ocak G. Rotmans J. I. Vossen C. Y. Rosendaal F. R. Krediet R. T. Boeschoten E. W. (2013). Type of arteriovenous vascular access and association with patency and mortality. BMC Nephrol. 14 (1), 79. 10.1186/1471-2369-14-79 23557085 PMC3621613

[B75] Padmanabhan J. Kinser E. R. Stalter M. A. Duncan-Lewis C. Balestrini J. L. Sawyer A. J. (2014). Engineering cellular response using nanopatterned bulk metallic glass. ACS Nano 8 (5), 4366–4375. 10.1021/nn501874q 24724817 PMC4046793

[B76] Peck M. Gebhart D. Dusserre N. McAllister T. N. L'Heureux N. (2012). The evolution of vascular tissue engineering and current state of the art. Cells Tissues Organs 195 (1-2), 144–158. 10.1159/000331406 21996786 PMC3325604

[B77] Pontiga F. Gaytán S. P. (2005). An experimental approach to the fundamental principles of hemodynamics. Adv. physiology Educ. 29 (3), 165–171. 10.1152/advan.00009.2005 16109796

[B78] Pradeep K. Lisa C. Priyamvada P. Yahya E. C. Viness P. (2019). Chapter 6 - nanoengineered biomaterials for vascular tissue engineering. Micro Nano Technol., 125–144.

[B79] Radke D. Jia W. Sharma D. Fena K. Wang G. Goldman J. (2018). Tissue engineering at the blood-contacting surface: A review of challenges and strategies in vascular graft development. Adv. Healthc. Mater. 7 (15), e1701461. 10.1002/adhm.201701461 29732735 PMC6105365

[B80] Riveros A. Garcia-Brand A. J. Rodriguez-Soto M. A. Sandoval N. Muñoz-Camargo C. Cruz J. C. (2022). Computational characterization of mechanical, hemodynamic, and surface interaction conditions: Role of protein adsorption on the regenerative response of TEVGs. Int. J. Mol. Sci. 23 (3), 1130. 10.3390/ijms23031130 35163056 PMC8835378

[B81] Rodriguez-Soto M. A. Suarez Vargas N. Riveros A. Camargo C. M. Cruz J. C. Sandoval N. (2021). Failure analysis of TEVG's I: Overcoming the initial stages of blood material interaction and stabilization of the immune response. Cells 10 (11), 3140. 10.3390/cells10113140 34831361 PMC8625197

[B82] Roh J. D. Sawh-Martinez R. Brennan M. P. Jay S. M. Devine L. Rao D. A. (2010). Tissue-engineered vascular grafts transform into mature blood vessels via an inflammation-mediated process of vascular remodeling. Proc. Natl. Acad. Sci. U. S. A. 107 (10), 4669–4674. 10.1073/pnas.0911465107 20207947 PMC2842056

[B83] Ruskowitz E. R. DeForest C. A. (2018). Photoresponsive biomaterials for targeted drug delivery and 4D cell culture. Nat. Rev. Mater. 3 (2), 17087. 10.1038/natrevmats.2017.87

[B84] Sadtler K. Wolf M. T. Ganguly S. Moad C. A. Chung L. Majumdar S. (2019). Divergent immune responses to synthetic and biological scaffolds. Biomaterials 192, 405–415. 10.1016/j.biomaterials.2018.11.002 30500722

[B85] Saito J. Kaneko M. Ishikawa Y. Yokoyama U. (2021). Challenges and possibilities of cell-based tissue-engineered vascular grafts. Cyborg Bionic Syst. 2021, 1532103. 10.34133/2021/1532103 36285145 PMC9494692

[B86] Sarkar S. Sales K. M. Hamilton G. Seifalian A. M. (2007). Addressing thrombogenicity in vascular graft construction. J. Biomed. Mater. Res. Part B, Appl. biomaterials 82 (1), 100–108. 10.1002/jbm.b.30710 17078085

[B87] Sarker M. D. Naghieh S. Sharma N. K. Chen X. (2018). 3D biofabrication of vascular networks for tissue regeneration: A report on recent advances. J. Pharm. Anal. 8 (5), 277–296. 10.1016/j.jpha.2018.08.005 30345141 PMC6190507

[B88] Shin'oka T. (2004). Clinical results of tissue-engineered vascular autografts seeded with autologous bone marrow cells. Nihon Geka Gakkai zasshi 105 (8), 459–463. 15373223

[B89] Shin'oka T. Imai Y. Ikada Y. (2001). Transplantation of a tissue-engineered pulmonary artery. N. Engl. J. Med. 344 (7), 532–533. 10.1056/NEJM200102153440717 11221621

[B90] Shokrani H. Shokrani A. Sajadi S. M. Seidi F. Mashhadzadeh A. H. Rabiee N. (2022). Cell-seeded biomaterial scaffolds: The urgent need for unanswered accelerated angiogenesis. Int. J. Nanomedicine 17, 1035–1068. 10.2147/IJN.S353062 35309965 PMC8927652

[B91] Song H. H. G. Rumma R. T. Ozaki C. K. Edelman E. R. Chen C. S. (2018). Vascular tissue engineering: Progress, challenges, and clinical promise. Cell Stem Cell 22 (3), 340–354. 10.1016/j.stem.2018.02.009 29499152 PMC5849079

[B92] Sridharan R. Cavanagh B. Cameron A. R. Kelly D. J. O'Brien F. J. (2019). Material stiffness influences the polarization state, function and migration mode of macrophages. Acta biomater. 89, 47–59. 10.1016/j.actbio.2019.02.048 30826478

[B93] Stejskalova A. Oliva N. England F. J. Almquist B. D. (2019). Biologically inspired, cell-selective release of aptamer-trapped growth factors by traction forces. Adv. Mater 31 (7), e1806380. 10.1002/adma.201806380 30614086 PMC6375388

[B94] Stolic R. (2013). Most important chronic complications of arteriovenous fistulas for hemodialysis. Med. Princ. Pract. 22 (3), 220–228. 10.1159/000343669 23128647 PMC5586732

[B95] Stoumpos S. Rankin A. Hall Barrientos P. Mangion K. McGregor E. Thomson P. C. (2021). Interrogating the haemodynamic effects of haemodialysis arteriovenous fistula on cardiac structure and function. Sci. Rep. 11 (1), 18102. 10.1038/s41598-021-97625-5 34518583 PMC8437985

[B96] Syedain Z. Reimer J. Lahti M. Berry J. Johnson S. Tranquillo R. T. (2016). Tissue engineering of acellular vascular grafts capable of somatic growth in young lambs. Nat. Commun. 7, 12951. 10.1038/ncomms12951 27676438 PMC5052664

[B97] Syedain Z. H. Graham M. L. Dunn T. B. O'Brien T. Johnson S. L. Schumacher R. J. (2017). A completely biological "off-the-shelf" arteriovenous graft that recellularizes in baboons. Sci. Transl. Med. 9 (414), eaan4209. 10.1126/scitranslmed.aan4209 29093182

[B98] Thamer M. Lee T. C. Wasse H. Glickman M. H. Qian J. Gottlieb D. (2018). Medicare costs associated with arteriovenous fistulas among US hemodialysis patients. Am. J. kidney Dis. 72 (1), 10–18. the official journal of the National Kidney Foundation. 10.1053/j.ajkd.2018.01.034 29602630

[B99] Traore M. A. George S. C. (2017). Tissue engineering the vascular tree. Tissue Eng. Part B Rev. 23 (6), 505–514. 10.1089/ten.teb.2017.0010 28799844 PMC5729878

[B100] Turner P. A. Thiele J. S. Stegemann J. P. (2017). Growth factor sequestration and enzyme-mediated release from genipin-crosslinked gelatin microspheres. J. biomaterials Sci. Polym. Ed. 28 (16), 1826–1846. 10.1080/09205063.2017.1354672 PMC5951619 28696181

[B101] Vahidkhah K. Diamond S. L. Bagchi P. (2014). Platelet dynamics in three-dimensional simulation of whole blood. Biophysical J. 106 (11), 2529–2540. 10.1016/j.bpj.2014.04.028 PMC4052243 24896133

[B102] Vajda J. Milojević M. Maver U. Vihar B. (2021). Microvascular tissue engineering-A review. Biomedicines 9 (6), 589. 10.3390/biomedicines9060589 34064101 PMC8224375

[B103] Vartanian S. M. Conte M. S. (2015). Surgical intervention for peripheral arterial disease. Circ. Res. 116 (9), 1614–1628. 10.1161/CIRCRESAHA.116.303504 25908732

[B104] Wang D. Wang X. Zhang Z. Wang L. Li X. Xu Y. (2019). Programmed release of multimodal, cross-linked vascular endothelial growth factor and heparin layers on electrospun polycaprolactone vascular grafts. ACS Appl. Mater. Interfaces 11 (35), 32533–32542. 10.1021/acsami.9b10621 31393107

[B105] Wang J. N. Kan C. D. Lin S. H. Chang K. C. Tsao S. Wong T. W. (2021). Potential of autologous progenitor cells and decellularized porcine artery matrix in construction of tissue-engineered vascular grafts. Organogenesis 17 (3-4), 72–84. 10.1080/15476278.2021.1963603 34405770 PMC9208767

[B106] Wang X. Chan V. Corridon P. R. (2022). Acellular tissue-engineered vascular grafts from polymers: Methods, achievements, characterization, and challenges. Polymers 14 (22), 4825. 10.3390/polym14224825 36432950 PMC9695055

[B107] Wang X. Chan V. Corridon P. R. (2022). Decellularized blood vessel development: Current state-of-the-art and future directions. Front. Bioeng. Biotechnol. 10, 951644. 10.3389/fbioe.2022.951644 36003539 PMC9394443

[B108] Wang Z. Cui Y. Wang J. Yang X. Wu Y. Wang K. (2014). The effect of thick fibers and large pores of electrospun poly(ε-caprolactone) vascular grafts on macrophage polarization and arterial regeneration. Biomaterials 35 (22), 5700–5710. 10.1016/j.biomaterials.2014.03.078 24746961

[B109] Wang Z. Liu C. Xiao Y. Gu X. Xu Y. Dong N. (2019). Remodeling of a cell-free vascular graft with nanolamellar intima into a neovessel. ACS Nano 13 (9), 10576–10586. 10.1021/acsnano.9b04704 31483602

[B110] Wautier J. L. Wautier M. P. (2013). Molecular basis of erythrocyte adhesion to endothelial cells in diseases. Clin. Hemorheol. Microcirc. 53 (1-2), 11–21. 10.3233/CH-2012-1572 22941965

[B111] Weinberg C. B. Bell E. (1986). A blood vessel model constructed from collagen and cultured vascular cells. Sci. (New York, NY) 231 (4736), 397–400. 10.1126/science.2934816 2934816

[B112] Wilhelmi M. Jockenhoevel S. Mela P. (2014). Bioartificial fabrication of regenerating blood vessel substitutes: Requirements and current strategies. Biomed. Eng./Biomed. Tech. 59 (3), 185–195. 10.1515/bmt-2013-0112 24583461

[B113] Wissing T. B. Bonito V. van Haaften E. E. van Doeselaar M. Brugmans M. Janssen H. M. (2019). Macrophage-driven biomaterial degradation depends on scaffold microarchitecture. Front. Bioeng. Biotechnol. 7, 87. 10.3389/fbioe.2019.00087 31080796 PMC6497794

[B114] Xenotransplantation (1997). Science, ethics, and public policy. ILAR J. 38 (1), 49–51.

[B115] Xu J. Ge H. Zhou X. Yan J. Chi Q. Zhang Z. (2005). Prediction of vascular tissue engineering results with artificial neural networks. J. Biomed. Inf. 38 (6), 417–421. 10.1016/j.jbi.2005.03.002 16337566

[B116] Yuan X. Li W. Yao B. Li Z. Kong D. Huang S. (2022). Tri-layered vascular grafts guide vascular cells' native-like arrangement. Polym. (Basel) 14 (7), 1370. 10.3390/polym14071370 PMC9003212 35406244

[B117] Zhang Y. Kumar P. Lv S. Xiong D. Zhao H. Cai Z. (2021). Recent advances in 3D bioprinting of vascularized tissues. Mater. Des. 199, 109398. 10.1016/j.matdes.2020.109398

